# Prevalence and new genotypes of *Enterocytozoon bieneusi* in sheltered dogs and cats in Sichuan province, southwestern China

**DOI:** 10.1051/parasite/2021029

**Published:** 2021-04-02

**Authors:** Yalin Zhong, Ziyao Zhou, Lei Deng, Haifeng Liu, Zhijun Zhong, Xiaoping Ma, Kun Zhang, Yingzhu Wang, Hualin Fu, Guangneng Peng

**Affiliations:** 1 The Key Laboratory of Animal Disease and Human Health of Sichuan Province, College of Veterinary Medicine, Sichuan Agricultural University Chengdu Sichuan 611130 PR China

**Keywords:** *Enterocytozoon bieneusi*, Dogs, Cats, ITS, China, Genotype

## Abstract

*Enterocytozoon bieneusi* is a common intracellular parasite that infects a wide range of hosts, including humans and companion animals, raising concerns of zoonotic transmission. However, there is limited epidemiological information on the prevalence and genotypes of *E. bieneusi* in sheltered dogs and cats in Sichuan province, southwestern China. A total of 880 fecal samples were collected from shelters in different cities of Sichuan province, including 724 samples from dogs, and 156 samples from cats. *Enterocytozoon bieneusi* was determined by sequence analysis of the ribosomal internal transcribed spacer (ITS). Overall, the prevalence of *E. bieneusi* was 18% (158/880), and the parasite was detected in 18.8% (136/724) and 14.1% (22/156) of the dogs and cats examined, respectively. Sequence analysis revealed the presence of five genotypes in dogs, including three known genotypes CD9 (*n* = 92), PtEb IX (*n* = 41), and Type IV (*n* = 1), and two novel genotypes SCD-1 (*n* = 1) and SCD-2 (*n* = 1). Similarly, four genotypes were identified in cats, including CD9 (*n* = 11), Type IV (*n* = 6), D (*n* = 4), and PtEb IX (*n* = 1). Genotypes D and Type IV have previously been identified in humans and are reported in sheltered dogs and cats in the present study, indicating that these animals could be as potential sources of human microsporidiosis infections.

## Abstract (fr)

*Enterocytozoon bieneusi* est un parasite intracellulaire commun qui infecte un large éventail d’hôtes, y compris les humains et les animaux de compagnie, ce qui soulève des problèmes de transmission zoonotique. Cependant, il existe peu d’informations épidémiologiques sur la prévalence et les génotypes d’*E. bieneusi* chez les chiens et les chats des refuges dans la province du Sichuan, au sud-ouest de la Chine. Au total, 880 échantillons de matières fécales ont été prélevés dans des refuges dans différentes villes de la province du Sichuan, dont 724 échantillons de chiens et 156 échantillons de chats. *Enterocytozoon bieneusi* a été déterminé par analyse de séquence de l’espaceur transcrit interne ribosomique (ITS). Dans l’ensemble, la prévalence d’*E. bieneusi* était de 18 % (158/880) et le parasite a été détecté chez 18,8 % (136/724) et 14,1 % (22/156) des chiens et des chats examinés, respectivement. L’analyse des séquences a révélé la présence de cinq génotypes chez le chien, dont trois génotypes connus CD9 (*n* = 92), PtEb IX (*n* = 41) et type IV (*n* = 1), et deux nouveaux génotypes SCD-1 (*n* = 1) et SCD-2 (*n* = 1). De même, quatre génotypes ont été identifiés chez les chats, dont CD9 (*n* = 11), Type IV (*n* = 6), D (*n* = 4) et PtEb IX (*n* = 1). Les génotypes D et de type IV ont été précédemment identifiés chez l’homme et sont rapportés chez des chiens et des chats des refuges dans la présente étude, ce qui indique que ces animaux pourraient être des sources potentielles d’infections par microsporidiose chez les humains.

## Introduction

*Enterocytozoon bieneusi*, an obligate intracellular parasite, is the most common microsporidian associated with humans and animals, posing a public health threat [14, 30]. The disease caused by *E. bieneusi* is commonly characterized by chronic or severe diarrhea in patients with compromized immune systems, such as acquired immune deficiency syndrome (AIDS) and organ transplantation recipients [5, 30]. However, some individuals infected with *E. bieneusi* do not exhibit any clinical symptoms, so they can represent carriers of this parasite and spread the spores to susceptible humans and animals [34]. Thus, the National Institute of Allergy and Infectious Diseases (NIAID) has listed *E. bieneusi* as a Category B Priority Pathogen (https://www.niaid.nih.gov/research/emerging-infectious-diseases-pathogens).

*Enterocytozoon bieneusi* isolates are usually characterized genetically by analysis of the internal transcribed spacer (ITS) region of the ribosomal RNA (rRNA) gene [31, 35]. To date, more than 500 distinct genotypes have been reported in humans and various animals [15]. Phylogenetic analysis has shown that all valid *E. bieneusi* ITS genotypes are clustered into 11 groups [14]. The genotypes in group 1, the largest group, have zoonotic potential, and group 2 is the second largest group and is mainly found in ruminants [14, 22]. The remaining groups, group 3 to group 11, contain genotypes that seem to be host-specific [7, 14].

Since *E. bieneusi* was first reported in a Haitian AIDS patient with severe diarrhea [4], and it has been identified in a broad range of wild and domestic animal hosts worldwide, including mammals (e.g. artiodactyls, carnivores, lagomorphs, perissodactyls, primates and rodents) and birds [3, 12, 25, 28, 36]. However, epidemiological data regarding the prevalence and genotypes of *E. bieneusi* in sheltered dogs and cats in Sichuan province are scarce. Stray dogs and cats infected with *E. bieneusi* could excrete the infectious spores, which increases the possibility of transmission between animals and humans, thereby posing a potential threat to public health. The purpose of this study was to determine the prevalence and genotypes of *E. bieneusi* in sheltered dogs and cats. Moreover, the present study also aimed to provide fundamental information for monitoring the transmission of microsporidiosis between humans and stray animals.

## Materials and methods

### Ethics statement

The present study protocol was reviewed and approved by the Research Ethics Committee and the Animal Ethics Committee of Sichuan Agricultural University. Permission was obtained from the shelter’s managers before the fecal specimens were collected.

### Collection of specimens

**Figure 1:**
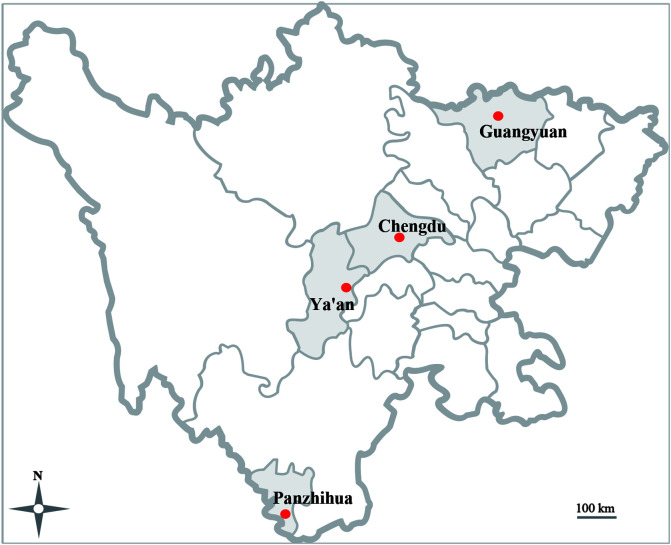
Geographical distribution of the sampled cities (filled red dot) in Sichuan Province.

During the period from September 2019 to June 2020, a total of 880 fecal specimens were collected from sheltered dogs and cats, including 724 samples from dogs, and 156 samples from cats. The sampling sites were distributed in different cities in Sichuan Province (Fig. 1). The stray dogs (around 8–12 dogs) were housed in the shared enclosure, so we only collected 3–4 fecal samples for each enclosure. The cats were kept in separate cages, and only one fecal sample was collected from each cat. Fecal samples were collected from the ground or bottom of cages using sterile gloves and then immediately placed into individual 15-mL sterile tubes with ice.

### DNA extraction and PCR amplification

Genomic DNA was extracted from each fecal sample (around 200 mg) using the E.Z.N.A.R^®^ Stool DNA kit (Omega Bio-tek Inc., Norcross, GA, USA), according to the manufacturer’s instructions. *Enterocytozoon bieneusi* was determined by nested PCR amplifying the ITS region of the rRNA gene using specific primers as described by Sulaiman et al. [27]. The PCR reaction volume (25 μL) contained 12.5 μL Taq PCR Master Mix (Sangon Biotech Co., Ltd., Shanghai, China), 1 μL each primer (0.4 μM), 2 μL genomic DNA, 1.5 mM MgCl_2_, and nuclease-free water to volume. Positive and negative controls were included in all the PCR reactions. The secondary PCR products were subjected to 1.5% agarose gel electrophoresis (AddGene, Watertown, MA, USA) and visualized by staining with SYBR Safe DNA Gel Stain (Thermo Fisher Scientific, Waltham, MA, USA).

### Nucleotide sequencing and data analysis

All secondary PCR products with the expected size (around 392 bp) were sequenced with primers used in the secondary PCR on an ABI 3730 XL DNA Analyzer. Nucleotide sequences obtained in the present study were aligned with each other and reference sequences downloaded from the GenBank database by using the program Clustal X 1.83 (http://www.clustal.org/). Genotypes of *E. bieneusi* isolates were grouped by phylogenetic analyses. All genotypes were identified based on 243 bp of the ITS region of *E. bieneusi* according to the established nomenclature system.

### Phylogenetic analysis

A neighbor-joining tree was constructed to assess the genetic relationships among the *E. bieneusi* genotypes acquired in this study and those published in previous studies, using the software Mega 6 (http://www.megasoftware.net/), and the evolutionary distances were calculated using the Kimura two-parameter model. The reliability of these trees was assessed by bootstrap analysis with 1000 replicates. The nucleotide sequences generated in the present study have been deposited in GenBank (https://www.ncbi.nlm.nih.gov/) under accession numbers MW464622 – MW464626 and MW464618 – MW464621 for dogs and cats, respectively.

### Statistical analysis

Data were analyzed using SPSS statistical software, version 22 (https://www.ibm.com/analytics/spss-statistics-software) and a Chi-square test was used to detect significant differences. A *p*-value < 0.05 was considered statistically significant. The adjusted odds ratio (OR) and 95% confidence interval (CI) for each variable were also calculated.

## Results

### Prevalence of *E. bieneusi* in sheltered dogs and cats

Overall, the prevalence of *E. bieneusi* was 18% (158/880), and the parasite was detected in 18.8% (136/724) and 14.1% (22/156) of the dogs and cats examined, respectively. In terms of the stray dogs, the prevalence of *E. bieneusi* among different locations ranged from 4.6% in Panzhihua and Guangyuan to 32% in Wenjiang (Table 1). However, the prevalences among different sources were not significantly different statistically (*p* > 0.05). As for stray cats, all tested areas found *E. bieneusi*-positive samples, with the highest prevalence in Shuangliu (15.3%, 13/85) (Table 2). The prevalence of *E. bieneusi* in Yaan and Panzhihua displayed consistent results, with 13% and 12.5%, respectively. Similarly, there was no statistically significant difference between different sources (*p* > 0.05).

**Table 1:** Prevalence and genotypes of *E. bieneusi* in sheltered dogs from different cities and sources in Sichuan province, southwestern China.

City	Source	No. examined	No. positive	Prevalence (%) (95% CI)	OR (95% CI)	*p-*value	Genotypes (*n*)
Chengdu	Shuangliu	158	16	10.1% (5.4–14.8)	Reference		CD9 (8); PtEb IX (7); SCD-1 (1)
	Wenjiang	250	80	32.0% (26.2–37.8)	4.176 (2.336–7.468)	0.000	CD9 (79); PtEb IX (1)
Ya’an	Yucheng	228	36	15.8% (11.1–20.5)	1.664 (0.888–3.117)	0.112	CD9 (4); PtEb IX (32)
Panzhihua	Dongqu	44	2	4.5% (−1.6–10.7)	0.423 (0.093–1.913)	0.264	CD9 (1); PtEb IX (1)
Guangyuan	Lizhou	44	2	4.5% (−1.6–10.7)	0.423 (0.093–1.913)	0.264	Type IV (1); SCD-2 (1)
Total		724	136	18.8% (15.9–21.6)			CD9 (92); PtEb IX (41); Type IV (1); SCD-1 (1); SCD-2 (1)

**Table 2:** Prevalence and genotypes of *E. bieneusi* in sheltered cats from different cities and sources in Sichuan province, southwestern China.

City	Source	No. examined	No. positive	Prevalence (%) (95% CI)	OR (95% CI)	*p-*value	Genotypes (*n*)
Chengdu	Shuangliu	85	13	15.3% (7.6–22.9)	Reference		CD9 (10); D (2); PtEb IX (1)
Ya’an	Yucheng	23	3	13.0% (−0.7–26.8)	0.831 (0.215–3.203)	0.788	CD9 (1); D (2)
Panzhihua	Dongqu	48	6	12.5% (3.1–21.9)	0.791 (0.280–2.237)	0.791	Type IV (6)
Total		156	22	14.1% (8.6–19.6)			CD9 (11); Type IV (6); D (4); PtEb IX (1)

### Genetic characterizations of *E. bieneusi*

A total of five genotypes were identified in stray dogs in the present study by sequence analysis of the nucleotide sequences of the ITS region of *E. bieneusi*, including three known genotypes (CD9, PtEb IX, and Type IV) and two novel genotypes named SCD-1 and SCD-2 (Table 1). Genotype CD9 was the most prevalent (92/136, 67.6%) and was observed in samples from all the three cities except for Guangyuan, followed by PtEb IX (30.1%, 41/136), which was detected in specimens from four locations. Genotypes Type IV, SCD-1, and SCD-2 were only found in one specimen, respectively (0.7%, 1/136).

Similarly, four genotypes were identified (CD9, Type IV, D, and PtEb IX) based on the analysis of the ITS sequences of the *E. bieneusi*-positive samples from stray cats (Table 2). Genotype CD9 was predominant, accounting for 50% (11/22). This was followed by genotype Type IV (27.3%, 6/22), which was detected in both Chengdu and Ya’an cities. Genotype D (18.2%, 4/22) was detected in both Chengdu and Ya’an cities. Genotype PtEb IX was only found in one specimen in Chengdu city (4.5%, 1/22).

### Phylogenetic analysis

**Figure 2:**
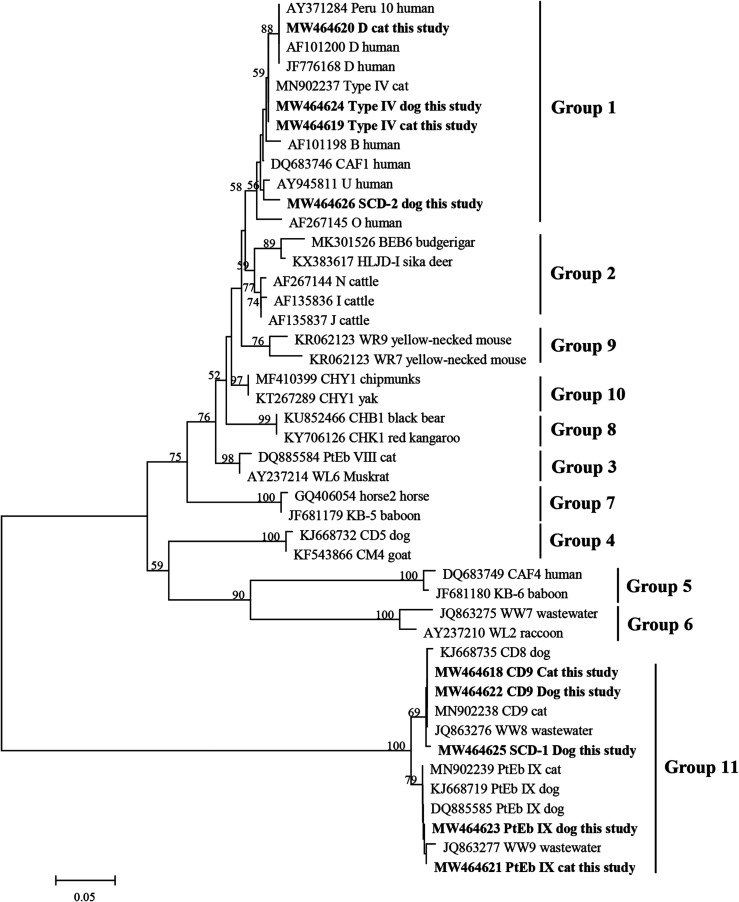
Phylogenetic relationship of *Enterocytozoon bieneusi* groups, the relationship between *E. bieneusi* genotypes obtained in the present study and other known genotypes deposited in GenBank was inferred by a neighbor-joining analysis of ITS sequences based on genetic distance by the Kimura-2-parameter model. The numbers on the branches represent percent bootstrapping values from 1000 replicates, with more than 50% shown in the tree. Each sequence is identified by its accession number, genotype, and host. Genotypes obtained in this study are showed in bold.

Phylogenetic analysis showed that all positive samples found in the present study belonged to two groups (group 1 and group 11). Genotypes D and Type IV were clustered into group 1 (Fig. 2). More importantly, the new genotype SCD-2 was also classified into the zoonotic group 1 (Fig. 2). Genotype CD9 and PtEb IX along with the new genotype SCD-1 were clustered into group 11 (Fig. 2).

## Discussion

In the present study, the overall prevalence of *E. bieneusi* in sheltered dogs and cats was 18% (158/880). Specifically, we found the prevalence of *E. bieneusi* in stray dogs was 18.8% (136/724), which was higher than that 8.6% (27/315) in dogs in eastern China [16], and in Shanghai, China 6% (29/485) [32]. In contrast, a more recent study in China revealed that the prevalence of *E. bieneusi* in dogs was 22.9% (149/651) [29]. In cats, 22 cats (14.1%) were found to be positive for *E. bieneusi*. The prevalence of *E. bieneusi* in cats in the present study was higher than that reported in previous studies in China, with ranges from 1.4% to 11.5% [10, 16]. The discrepancy in *E. bieneusi* prevalence may be associated with the fact that the fecal samples detected in the present study included those from sheltered dogs and cats, which lived in poor living conditions. Importantly, it has been determined that the poor living conditions were a major risk factor for contracting *E. bieneusi* infections [29].

In addition to China, *E. bieneusi* has been reported in dogs in various countries, with prevalence varying greatly. For example, the prevalence of *E. bieneusi* in stray dogs in Iran was the lowest, only 0.8% (2/237) [1]. Similarly, low *E. bieneusi* prevalence was also observed in dogs in Japan (4.4%, 26/597) [19], and in Poland (4.9%, 4/82) [20]. While high *E. bieneusi* prevalence was reported in a different study in Iran (25.8%, 8/100) [8], up to 100% prevalence was recorded in Portugal and the United States [6, 17]. Several studies have determined that the prevalence of *E. bieneusi* in cats was also very different. Relatively low prevalence was found in Brazil (3.3%, 2/60) [21], and in the Czech Republic (2.5%, 3/118) [11]. In contrast, higher prevalence was reported in Colombia (17.4%, 8/46) [26], and in Portugal (100%, 6/6) [17]. However, we need to be cautious when interpreting this huge discrepancy, because many studies only included limited samples.

By sequence analysis of the ITS region of the rRNA of *E. bieneusi*, five genotypes were identified out of 136 *E. bieneusi* isolates from stray dogs, including three known and two novel genotypes (Table 1). Genotype CD9 showed predominance, accounting for 67.6% (92/136). Genotype CD9 was also reported in dogs and cats in southern China [29]. Genotype PtEb IX was the second most common in the present study, which was also the predominant genotype in dogs in various countries [10, 18, 19, 24]. Genotype Type IV has been identified in dogs in China and Colombia [10, 24]. Interestingly, two novel genotypes (SCD-1 and SCD-2) were identified for the first time in stray dogs, suggesting genetic variability of *E. bieneusi* to adapt to different host gut environments.

Similarly, four known genotypes were identified in stray cats in the present study. CD9 was the most common genotype in cats as well (50%, 11/22). In a previous study, CD9 has been identified in household cats in Guangzhou province, southern China [29]. Type IV and D were the most common genotypes in cats in previous studies [10, 11, 16, 21, 32]. Genotypes D and Type IV are well known as zoonotic genotypes and have a broad-host range [23, 33]. They have been reported in a wide range of animals, including non-human primates [9], chipmunks [2], captive wildlife [12], and domestic animals [13]. In this study, genotype PtEb IX was found in only one fecal sample, which is consistent with a previous study in cats in China [10]. However, genotype PtEb IX was the most common in cats in another study [29]. The discrepancy in prevalence of different genotypes may be related to geographical differences.

## Conclusion

To the best of our knowledge, this is the first report of the prevalence and genotypes of *E. bieneusi* in sheltered dogs and cats in Sichuan province. The prevalence of *E. bieneusi* was 18.8% (136/724) and 14.1% (22/156) in dogs and cats, respectively. Zoonotic genotypes D and Type IV have been identified in humans and were also reported in sheltered dogs and cats, implying that these animals infected with *E. bieneusi* may pose a potential threat to human health.
